# Epidural analgesia, neonatal care and breastfeeding

**DOI:** 10.1186/s13052-014-0082-6

**Published:** 2014-11-29

**Authors:** Antonio Alberto Zuppa, Giovanni Alighieri, Riccardo Riccardi, Maria Cavani, Alma Iafisco, Francesco Cota, Costantino Romagnoli

**Affiliations:** Department of Pediatric, Division of Neonatology, “A. Gemelli” General Hospital, Largo Agostino Gemelli, 8, 00168 Roma, RM Italy

## Abstract

The objective of our study is to evaluate the correlation between epidural analgesia during labor, start of breastfeeding and type of maternal-neonatal care.

Two different assistance models were considered: *Partial* and *Full Rooming-in*.

In this cohort study, 2480 healthy infants were enrolled, 1519 in the *Partial Rooming-in* group and 1321 in the *Full Rooming-in group*; 1223 were born to women subjected to epidural analgesia in labor.

In case of *Partial Rooming-in* the rate of exclusive or prevailing breastfeeding is significant more frequent in newborns born to mothers who didn't receive analgesia. Instead, in case of *Full Rooming-in* the rate of exclusive or prevailing breastfeeding is almost the same and there's no correlation between the use or not of epidural analgesia.

The good start of lactation and the success of breastfeeding seems to be guaranteed by the type of care offered to the couple mother-infant, that reverses any possible adverse effects of the use of epidural analgesia in labor.

## Background

Breastfeeding provides optimal nutrition for infants and improves maternal health. Some studies attribute to breastmilk the reduction of the frequency and severity of neonatal infectious diseases and a protective function against sudden infant death syndrome, diabetes, lymphomas, allergies, and chronic digestive diseases. Lactation explicates several benefits for mothers because it seems to reduce the incidence of postpartum bleeding and the risk of ovarian and breast cancer, it favors the return to pre-pregnancy body weight and improves bone remineralization [1,2].

Therefore the American Academy of Pediatrics (AAP) recommends breastfeeding for almost the first six months of life [3].

In recent years there was an increase of epidural analgesia for pain management during labor. Several studies tried to find an association between epidural analgesia and breastfeeding. Most of these studies are retrospective, observational and nonrandomized, and the confounding variables make the results unreliable and sometimes conflicting [4-10].

Recently some authors focused their studies on type of assistance given to newborns, analyzing mother-child relationship during the first days of life, and they don't consider epidural analgesia a risk factor for breastfeed [2,11-16].

The objective of our study was to evaluate the correlation between epidural analgesia during labor, start of breastfeeding and type of maternal-neonatal care. Primary outcome was the type of feeding assessed from the 48th to the 72nd hour of life, we also evaluated the influence of labor analgesia on neonatal Apgar score.

## Methods

In this cohort retrospective study conducted for a period of two years, 2840 healthy infants born at the ‘A. Gemelli’ General Hospital (Rome) with gestational age (GA) ≥37 weeks were eligible. They were delivered vaginally after uncomplicated pregnancies. Most of the mothers were middle-class and graduated. Exclusion criteria were Apgar score <7 at 1 or 5 min and high risk pregnancy. Two different assistance models were considered to encourage and support breastfeeding by all eligible mothers:

- Partial Rooming-in (PR-I): newborns were in mother's room from 10 am to 8 pm, with the occasional presence of the nurse. At night newborns were transferred and assisted in the nursery to offer the necessary care by the caregivers.
- Full Rooming-in (FR-I): newborns and mothers were together all day long, with continuous assistance of the nursing staff, to make the neonatal-care easier for the mother and to assess the appropriateness of breastfeeding. The access to full rooming-in was possible only if it was available an appropriate room, without planning in advance [17].

Nowadays epidural analgesia is implemented in refracted bolus or in continuous infusion combining local anesthetics (Bupivacaine or Ropivacaine) with opioids (Fentanyl or Sufentanil) [18].

In our hospital laboring women received epidural analgesia with an initial bolus 18–20 ml of Sufentanil 10 μg plus Ropivacaine 0.10%, until reaching analgesic block and repeated bolus of Ropivacaine 0.15-0,2% of 8–10 ml every two hours, to maintain the analgesic block. The initial bolus was given only if there were painful and effective contractions, of appropriate intensity and frequency, and if the fetal head was at the level of the uterine cervix.

Type of feeding was assessed from the 48th to the 72nd hour of hospitalization of the newborns involved in the study. Type of feeding was distinguished, according to WHO-guidelines [19] in:

- Exclusive Breastfeeding (EB): infant received only breast milk from mother and no other liquids or solids, with the exception of drops or syrups containing vitamins, mineral supplements or medicines;
- Prevalent Breastfeeding (PB): Newborns were mostly breastfed, but they could also have received water or other water-based liquids, (sweetened and flavored water teas, infusions);
- Mixed Feeding (MF): Infants received formula as integration to maternal milk;
- Artificial Feeding (AF): Newborns did not receive breast milk but only formula.

All data were retrospectively collected and stored on a database. Continuous variables were presented as mean ± standard deviation (SD) and categorical variables as percentage. Comparisons with the variables were performed using Student’s T-test in case of normal distribution. Significance was accepted at p < 0.05. Statistical analysis was performed using the software Graph Pad 4.

A logistic regression was performed to evaluate the risk “mixed or artificial feeding in infants between 48th and 72nd hour of life” with regard to the independent variables: mother’s parity, assistance model (partial or full rooming-in) and use of epidural analgesia during labor. The findings are presented as odds ratio (OR) with 95% confidence intervals (CI), standard errors and p-values. The analyses were performed using the “Stata Statistical Software: Release 10” (StataCorp LP, College Station, Tx). In our research study we have got the ethical approval by the ethical committee of the Catholic University of the Sacred Heart of Rome.

## Results

2840 term newborns, by vaginal childbirth have been enrolled: 1617 were born to women not subject to epidural analgesia in labor (*No Analgesia* group); 1223 were born to mothers subjected to epidural analgesia in labor (*Epidural Analgesia* group). There were no differences between the groups studied with respect to maternal age, gestational age and parity (Table 1).

**Table 1:** **Demographic characteristics**

	***No analgesia group***			***Epidural analgesia group***			***P value***
**Gestational age (weeks)**	**mean ± ds**	39,1 ± 1,01		**mean ± ds**	39,4 ± 3,59		Ns*
	**range**	37 – 41		**range**	37 – 41		
**Maternal age (aa)**	**mean ± ds**	32,9 ± 6,3		**mean ± ds**	32,4 ± 5,5		Ns*
	**range**	20 – 45		**range**	18 – 44		
**Parity**		**N°**	**%**		**N°**	**%**	
	**Primiparous**	885	54.8	**Primiparous**	680	55.6	Ns*
	**Multiparous**	732	45.2	**Multiparous**	543	44.4	

*Ns= not significant.

Apgar score did not show any difference between the two groups, nor at one minute or at five minutes of life.

The newborns studied received milk from the 48th hour to the 72nd hour of life as follows: 2024 (71,3%) newborns received exclusively or predominantly breastmilk, 801(28,2%) received breastmilk with integration of formula, 15 (0,6%) were fed exclusively with formula.

Considering the use or not of epidural analgesia, the rates of newborns exclusive or prevailing breastfed are higher in the first group (73,5% in "no Analgesia group” vs 68,3% in “Analgesia group”, *p* = 0,002). Mixed feeding is less frequent than exclusive or prevailing breastfeeding but its prevalence is higher in the second group (25,9% in "no Analgesia group” vs 31,2% in “Analgesia group”, *p* = 0,002) (Table 2).

**Table 2:** **Type of feeding in the two studied groups**

***Type of feeding***	***No analgesia***	***Epidural analgesia***	***P value***
***AF***	9 (0,6%)	6 (0,5%)	Ns*
***MF***	419 (**25,9%**)	382 (**31,2%**)	0,002
***EB o PB***	1189 (**73,5%**)	835 (**68,3%**)	0.002

*Ns= not significant.

*Partial Rooming-in* was the assistance model for 1519 newborns (53,5%) and *Full Rooming-in* for 1321 (46,5%). Prevalence of breastfeeding was higher in Full than in Partial Rooming-in (92,2% vs 53,1) (Table 3).

**Table 3:** **Type of feeding and assistance model**

***Type of feeding***	***Partial rooming-in***	***Full rooming-in***
***AF***	0,8%	0,2%
***MF***	46,1**%**	7,6**%**
***EB o PB***	53,1**%**	92,2**%**

Stratifying infants by model of assistance and differentiating between the two groups “No Analgesia” vs “Epidural Analgesia” we note that in case of *Partial Rooming-in* the rate of exclusive or prevailing breastfeeding is significant more frequent in newborns born to mothers who didn't receive analgesia (*p* <0,001; 59,2% vs 44,1%). Instead, in case of *Full Rooming-in* the rate of exclusive or prevailing breastfeeding is almost the same and there's no correlation between the use or not of epidural analgesia (*p* <0,001; 91,7% vs 92,8%) (Tables 4 and 5).

**Table 4:** **Type of feeding in the two studied groups in partial rooming-in assistance**

***Type of feeding***	***No analgesia***	***Epidural analgesia***	***P value***
***AF***	8 (0,9%)	4 (0,7%)	Ns*
***MF***	361 (**39,9%**)	340 (**55,3%**)	< 0.001
***EB o PB***	535 (**59.2%**)	271 (**44,1%**)	< 0.001

*Ns= not significant.

**Table 5:** **Type of feeding in the two studied groups in full rooming-in assistance**

***Type of feeding***	***No analgesia***	***Epidural analgesia***	***P value***
***AF***	1 (0,1%)	2 (0,3%)	Ns*
***MF***	58 (**8,1%**)	42 (**6.9%**)	Ns*
***EB o PB***	654 (**91,7%**)	564 (**92,8%**)	Ns*

*Ns= not significant.

### Multivariable analyses

In partial rooming-in assistance, newborn were more likely to have received mixed or artificial feeding between the 48^th^ and the 72^nd^ hour of life (OR 10.99, p < 0.0001, 95% CI 8.76-13.79). Mixed or artificial feeding between the 48^th^ and the 72^nd^ hour of life is also associated, but to a lesser extent, with maternal use of epidural analgesia during labor (OR 1.61, p < 0.0001, 95% CI 1.34-1.94). Maternal parity, instead, is not associated with type of newborn feeding (OR 0.99, p > 0.05, 95% CI 0.78-1.08) (Table 6).

**Table 6:** **Multivariable analyses to evaluate risk of “mixed or artificial feeding in infants between 48** ^**th**^ **and 72** ^**nd**^ **hour of life” with regard to independent variables considered**

***Comparison***	***Odds ratio***	***95% confidence interval***	***P value***
***Partial rooming-in vs full rooming-in***	10.99	8.76 – 13.79	0.0001
***Epidural analgesia vs no analgesia***	1.61	1.34 – 1.94	0.0001
***Primiparous vs multiparous***	0.99	0.78 – 1.08	Ns*

*Ns= not significant.

## Discussion and conclusion

To minimize the maternal discomfort and its effects for the fetus, in recent years the use of epidural analgesia during labor is increased. The objective of this study was to determine whether epidural analgesia interfere with the start of breastfeeding in the first days of life.

The effects that drugs used in epidural analgesia may cause to fetus can be direct, which are rare and especially due to overdose of the drugs used, and indirect, linked to the physiological and biochemical changes that they determine in the mother that may indirectly affect the fetus and newborn.

As evidenced by Halpern et al. the use of epidural opioid analgesia is associated with greater incidence of higher Apgar scores at 1 'and 5' and to a lesser need of neonatal resuscitation and use of Naloxone than with systemic opioid analgesia [2]. Some authors have focused on the influence of epidural analgesia on starting breastfeeding.

The results of many studies in recent years are largely conflicting. First of all, the problem was whether the reduction of pain with epidural analgesia could affect breastfeeding. Some studies suggested that this is associated with a delayed start of lactation and to an earlier suspension, others have found no association. Halpern and co-workers in their observational study reported that analgesia in labor is not associated with a reduction of breastfeeding in their study population at 6–8 weeks postpartum [2,20]. In their work, rather, it is highlighted the importance of the promotion of breastfeeding in hospital management, as reaffirmed in 2002 by Leighton [21].

In our hospital there is a constant effort to promote breastfeeding, supporting early mother-infant relationship, increasing the rooming-in and the breastfeeding on demand.

Even Albani and colleagues in 1999 reported the absence of adverse effects of epidural analgesia on breastfeeding, with the same rate of breastfeeding at 1, 4, 6 weeks post-partum in case of use or not of epidural analgesia [22].

In a study of 2010 Reynolds and co-workers concluded that while epidural analgesia in labor may be associated with some short-term side effects, its effects on the child, when compared with systemic analgesia, are better for Apgar scores, acid–base balance and breastfeeding [13]. Beilin and colleagues performed the only randomized controlled trial to evaluate the dose-dependent effect of epidural fentanyl on breastfeeding success [23]. They showed that epidural analgesia with fentanyl at high dosage (>150 μg) associated with local anesthetics, compared with epidural analgesia with lesser amounts of fentanyl, is more often associated with stopping breastfeeding before 6 weeks of life.

Wilson and colleagues, in their randomized controlled trial, confirmed that women who did not receive epidural analgesia did not show higher rates of breastfeeding compared with women receiving epidural analgesia. In fact, there is a lower rate of breastfeeding in women not undergoing epidural analgesia but pethidine systemically [20].

In our study we have observed that the use of epidural analgesia does not adversely affect the Apgar score at one or five minutes of life: there are values comparable between the groups. As regards breastfeeding, we considered the women who breastfed their children between 48th and 72nd hour of life. We noted that, on the general population, there is a prevalence statistically significant of breastfeeding in the group of women who didn’t undergo epidural analgesia. This result, however, is strongly due to the assistance model used. It's important to analyze type of feeding in relation to the type of hospitalization model, total or partial Rooming-in. In the first case there was a prevalence of exclusively or predominantly breastfeeding in both groups. Instead, in case of partial Rooming-in, we see a prevalence of exclusively or predominantly breastfeeding in the group of mother who didn’t undergo epidural analgesia and this was statistically significant.

Although multivariate analysis indicates the use of epidural analgesia as an independent factor which interferes on breastfeeding, the protective effect of the type of rooming in seems to be very high and higher than the risk determined by epidural analgesia itself, but these results should be confirmed on other surveys.

As stated by Wieczorek and Pandya in their recent studies [24,25], we believe that the difference in the type of feeding is not due to the influence of analgesia used but rather to the type of Rooming-in carried out, total or partial, and to the clinical monitoring necessary to neonatal well-being. It seems, essential to adopt a model of care that favors an early mother-infant relationship and ensure its continuation with total Rooming-in.

Therefore, in *full rooming-in*, with an early and continuous mother-infant contact, there are no differences in the type of breastfeeding among the group of infants born to mothers who underwent epidural analgesia and the group of newborns born to mothers not subjected to analgesia. Furthermore if we consider only the group of infants born to mother that underwent epidural analgesia there is a high prevalence of exclusive or prevailing breastfeeding in *full rooming-in* model assistance compared with *partial rooming-in*, with a frequency of 92.8% vs 44.1%.

We can conclude that the good start of lactation and the success of breastfeeding seems to be guaranteed by the type of care offered to the couple mother-infant, that reverses any possible adverse effects of the use of epidural analgesia in labor.
